# Diagnostic and Management Challenges in Mixed-Source MDR Sepsis in a Kidney Transplant Recipient with an Infected Sacral Pressure Ulcer Treated with VAC Therapy: A Case Report

**DOI:** 10.3390/diagnostics16203263

**Published:** 2026-10-09

**Authors:** Bogdan Mihnea Ciuntu, Alexandra-Simona Zamfir, Lili Gabriela Lozneanu, Tanevski Adelina, Stefan Lucian Toma, Carmen Lacramioara Zamfir, Rares Stefan Costarnache, Florinel Cosmin Bida, Mihai Bogdan Vascu, Camelia Tamas

**Affiliations:** 1 Faculty of Medicine, “Grigore T. Popa” University of Medicine and Pharmacy, 700115 Iasi, Romania; bogdan-mihnea.ciuntu@umfiasi.ro (B.M.C.); camelia.tamas@umfiasi.ro (C.T.); 2 Department of General Surgery, County Clinical Emergency Hospital St. Spiridon, 700111 Iasi, Romania; 3 Department of Medical Sciences III, Faculty of Medicine, “Grigore T. Popa” University of Medicine and Pharmacy, 700115 Iasi, Romania; 4 Regional Institute of Oncology, 700483 Iasi, Romania; 5 Faculty of Materials Science and Engineering, Gheorghe Asachi Technical University of Iasi, 700050 Iasi, Romania; 6 Department of Morpho-Functional Sciences I, Faculty of Medicine, “Grigore T. Popa” University of Medicine and Pharmacy, 700115 Iasi, Romania; carmen.zamfir@umfiasi.ro; 7 Faculty of Dental Medicine, “Grigore T. Popa” University of Medicine and Pharmacy, 700115 Iasi, Romania; cosmin.bida@umfiasi.ro (F.C.B.); mihai.vascu@umfiasi.ro (M.B.V.); 8 Department of Plastic Surgery, “Grigore T. Popa” University of Medicine and Pharmacy, 700115 Iasi, Romania

**Keywords:** kidney transplantation, diagnostic challenges, mixed-source sepsis, multidrug-resistant organisms, sacral pressure ulcer, urinary tract infection, negative pressure wound therapy, vacuum-assisted closure, renal allograft preservation

## Abstract

**Background**: Kidney transplant recipients are particularly vulnerable to severe infections because of chronic immunosuppression, impaired wound healing, repeated healthcare exposure, and the potential nephrotoxicity of antimicrobial therapy. We report a complex case of multidrug-resistant (MDR) sepsis in a kidney transplant recipient with two concomitant potential infectious sources: an infected sacral pressure ulcer and recurrent urinary tract infection. **Case Presentation**: A 31-year-old kidney transplant recipient developed traumatic paraplegia following a road traffic accident. Prolonged immobilization was complicated by a progressive sacral pressure ulcer and recurrent urinary tract infections in the setting of neurogenic bladder dysfunction. During hospitalization, wound and urinary cultures identified MDR Gram-negative organisms, including *Klebsiella pneumoniae*, *Acinetobacter baumannii*, and *Proteus mirabilis*. Blood cultures obtained during systemic deterioration were positive for *K. pneumoniae*. **Diagnostic Assessment and Therapeutic Intervention**: Severe systemic infection developed with hemodynamic deterioration and markedly elevated inflammatory biomarkers. Because both the urinary tract and infected sacral wound represented plausible infectious reservoirs, the primary source of bacteremia could not be established unequivocally. Management required extensive surgical debridement, negative pressure wound therapy with instillation, culture-directed antimicrobial therapy, and close monitoring of renal allograft function. Gentamicin was used as a culture-directed therapeutic option in the setting of limited antimicrobial alternatives and impaired renal function. **Conclusions**: This case illustrates the diagnostic and therapeutic complexity of severe MDR infection in kidney transplant recipients when multiple potential infectious sources coexist. Successful management required early source control, microbiological assessment, individualized antimicrobial selection, and multidisciplinary monitoring aimed at controlling infection while preserving renal allograft function.

## 1. Introduction

Pressure ulcers remain a significant clinical challenge, particularly in patients with prolonged immobilization, such as those with spinal cord injuries. These lesions are associated with substantial morbidity, prolonged hospitalization, and increased healthcare costs. In severe cases, deep pressure ulcers may progress to extensive soft tissue infection, osteomyelitis, and systemic complications, including sepsis [1].

The risk and severity of pressure ulcers are markedly increased in immunocompromised patients, especially in solid organ transplant recipients receiving long-term immunosuppressive therapy [2]. In this population, impaired immune response, delayed wound healing, and increased susceptibility to opportunistic and multidrug-resistant (MDR) infections contribute to a more aggressive clinical course [3]. Renal transplant recipients represent a particularly vulnerable subgroup, as both severe infection and its treatment may directly impact graft function [4]. Severe infections and sepsis episodes in solid organ transplant recipients are associated with high morbidity and mortality, necessitating early recognition and highly industrialized therapeutic management [4].

Infectious complications in such patients are often complex and polymicrobial, frequently involving MDR pathogens such as *Klebsiella pneumoniae* and *Acinetobacter baumannii* [5]. The therapeutic management becomes especially challenging when antimicrobial susceptibility is limited to agents with known nephrotoxic potential, such as aminoglycosides [6]. This creates a critical clinical dilemma between preserving renal graft function and ensuring adequate infection control to prevent life-threatening progression [6].

Sepsis originating from pressure ulcers, although less commonly reported compared to other sources, represents a severe and potentially fatal complication, particularly in immunosuppressed individuals. When associated with additional infectious foci, such as recurrent urinary tract infections, the clinical picture may evolve rapidly toward septic shock, requiring prompt recognition and aggressive multidisciplinary management [1].

Negative pressure wound therapy (NPWT), commonly referred to as vacuum-assisted closure (VAC), has emerged as an effective adjunct in the management of complex soft tissue defects, promoting wound drainage, reducing bacterial load, and stimulating granulation tissue formation [7,8]. However, successful outcomes in critically ill and immunocompromised patients depend not only on local wound management but also on timely and appropriate systemic antimicrobial therapy.

Although MDR infections and pressure-ulcer-associated infections are individually well recognized in immunocompromised patients, published clinical descriptions addressing their coexistence with recurrent urinary tract infection in kidney transplant recipients remain limited. In particular, there is limited evidence illustrating antimicrobial decision-making when severe MDR infection requiring systemic therapy occurs in a patient with impaired function of a solitary renal allograft.

We present the case of a young renal transplant recipient who developed severe infectious complications following traumatic paraplegia, culminating in sepsis of a mixed origin. This case highlights the complexity of managing MDR infections in immunosuppressed patients and underscores the importance of a multidisciplinary approach and individualized therapeutic decision-making, particularly when life-saving treatment may pose a risk to graft function [9,10].

## 2. Case Presentation

### 2.1. Patient Information and Initial Presentation

A 31-year-old man with a history of kidney transplantation for end-stage kidney disease secondary to a congenital solitary kidney was admitted acutely in January 2025 following a severe road traffic accident that resulted in traumatic T10 paraplegia. Kidney transplantation had been performed 5 years before the current admission. Before the traumatic event, renal allograft function had remained remarkably stable, with a documented baseline serum creatinine of approximately 1.2 mg/dL maintained consistently over the preceding 6 months during routine outpatient check-ups. The renal allograft represented his only functioning renal unit, and the patient was receiving long-term maintenance immunosuppressive therapy. This clinical background placed him at an increased risk of opportunistic infections and impaired wound healing.

The traumatic paraplegia required prolonged immobilization and was associated with neurogenic lower urinary tract dysfunction. Prior to this traumatic accident, the patient’s chronic bladder emptying baseline at home was managed via clean intermittent self-catheterization. However, during the initial inpatient period immediately following the trauma, urinary output had to be closely monitored in the hospital through an indwelling Foley catheter for 72 h to ensure precise fluid volume surveillance. After acute post-injury stabilization and subsequent catheter removal, clean intermittent self-catheterization was resumed as the standard maintenance regimen because spontaneous and complete bladder emptying could not be achieved.

At the time of his initial acute presentation to the emergency department, the serum creatinine level was 2.28 mg/dL, corresponding to an estimated glomerular filtration rate (eGFR) of 36.85 mL/min/1.73 m^2^ calculated via the 2021 CKD-EPI equation. Compared to his stable outpatient baseline, this acute rise represented a clinically significant Acute Kidney Injury (AKI stage 2), secondary to post-traumatic hypovolemia and acute neurogenic urinary retention.

Preventive pressure ulcer measures, including regular repositioning, pressure redistribution surfaces, nutritional support, and daily skin inspection, were implemented according to institutional protocols. Nevertheless, pressure injuries may still occur despite appropriate preventive measures in patients with multiple major risk factors. In this patient, prolonged immobilization, neurological impairment, chronic immunosuppression, and the overall clinical burden contributed to the development and subsequent progression of the sacral pressure ulcer.

### 2.2. Clinical Course and Laboratory Findings

Over the following approximately three months, the patient experienced a complex and prolonged clinical course. Recurrent urinary tract infections required repeated courses of antimicrobial therapy and additional hospital care. Simultaneously, the sacral pressure ulcer became progressively infected, with increasing local inflammation, necrosis, and wound extension, raising concern for a polymicrobial soft tissue infection.

Nutritional support was aggressively optimized by implementing a high-protein, hypercaloric regimen supplemented with arginine, zinc, and antioxidants to promote tissue repair in the stage IV ulcer. Specific glutamine-based immunonutrition was deliberately omitted, in strict accordance with clinical guidance for metabolic management during active severe sepsis [11].

The clinical course was further complicated by a secondary Clostridioides difficile infection, associated with prolonged broad-spectrum antimicrobial exposure. The diagnosis was established via a fecal stool PCR assay demonstrating positive results for genes encoding toxins A and B. The infection was managed and resolved using a 10-day course of oral vancomycin (125 mg every 6 h), achieving complete resolution of symptoms.

Microbiological investigations performed on wound secretions and urine specimens yielded multiple multidrug-resistant organisms, including *Klebsiella pneumoniae*, *Acinetobacter baumannii*, and *Proteus mirabilis*.

The microbiological profile reflected the cumulative impact of prolonged hospitalization, immunosuppression, repeated antimicrobial exposure, urinary tract instrumentation, and chronic wound evolution. Importantly, the subsequent septic episode occurred in a clinical context where both urinary and wound-related microbial reservoirs had to be considered, requiring correlation between microbiological findings and the clinical syndrome rather than interpretation of culture results in isolation.

The clinical evolutionary trajectory was further complicated by a secondary Clostridioides difficile infection, precipitated by the repeated and prolonged pressure of systemic broad-spectrum antimicrobial therapies. The diagnosis of this complication was formally confirmed via an automated fecal stool PCR assay demonstrating positive results for genes encoding toxins A and B. The infection was targeted and successfully eradicated using a 10-day course of oral Vancomycin administered at a dose of 125 mg every 6 h, achieving full resolution of diarrheal symptoms.

Despite empirical antibiotic treatment, the patient’s clinical condition progressively deteriorated. He developed a persistent systemic inflammatory response, worsening general status, and severe anemia requiring repeated blood transfusions. Serial laboratory investigations demonstrated substantial fluctuations in hemoglobin and hematocrit levels, together with impaired renal allograft function and increasing inflammatory activity.

At admission, the serum creatinine level was 2.28 mg/dL, corresponding to an estimated glomerular filtration rate of 36.85 mL/min/1.73 m^2^. Hemoglobin progressively decreased from 8.3 g/dL on Day 1 to 5.4 g/dL at the time of sepsis diagnosis, while C-reactive protein increased from 5.2 mg/L to 31.2 mg/L during the subsequent clinical course. White blood cell count, platelet count, procalcitonin, and presepsin were serially monitored throughout hospitalization. At the time of sepsis diagnosis, procalcitonin reached 77.49 ng/mL and presepsin 2026 pg/mL, findings consistent with severe bacterial sepsis.

The complete integration of clinical milestones, microbiological isolation windows, targeted antimicrobial adjustments, and the longitudinal trajectory of hematological, inflammatory, and renal biomarkers throughout the hospitalization course is comprehensively synthesized within Table 1.

### 2.3. Diagnostic Assessment

From a diagnostic perspective, the infectious course was interpreted in two sequential phases. The first phase was dominated by progressive local infection of the sacral pressure ulcer and required surgical source control and wound management. The second phase was characterized by acute systemic deterioration and *Klebsiella pneumoniae* bacteremia in the setting of recurrent urinary tract infection and a recently treated infected sacral wound. Because both anatomical sites represented plausible infectious reservoirs, the primary source of the bloodstream infection could not be established with certainty.

The diagnostic assessment was particularly challenging because several potential infectious sources coexisted in an immunosuppressed patient with pre-existing renal allograft dysfunction. The patient had neurogenic bladder requiring repeated urinary tract instrumentation, a history of recurrent urinary tract infections, and an extensively infected sacral pressure ulcer containing necrotic tissue. Prolonged hospitalization, repeated exposure to broad-spectrum antibiotics, chronic immunosuppression, and intermittent self-catheterization further increased the probability of infection with multidrug-resistant organisms.

Systemic infection was suspected following progressive clinical deterioration. According to the Sepsis-3 consensus definition, the diagnosis of bacterial sepsis is established by documented or strongly suspected infection combined with an acute change in the total Sequential Organ Failure Assessment (SOFA) score of 2 points or more, representing acute infection-induced multi-organ dysfunction. In this patient, the clinical syndrome met the criteria for true bacterial sepsis, presenting with a calculated acute SOFA score of 5 points at the time of systemic deterioration. This score was documented by three distinct system dysfunctions: 2 points for cardiovascular failure characterized by persistent arterial hypotension of 80/60 mmHg requiring transient vasopressor support with intravenous Noradrenaline titrated up to 0.05 mcg/kg/min to maintain a mean arterial pressure above 65 mmHg; 2 points for acute renal dysfunction manifested by a serum creatinine of 2.28 mg/dL, representing a significant acute increase over his stable chronic outpatient baseline of 1.20 mg/dL; and 1 point for hematological dysfunction documented by an acute sepsis-induced thrombocytopenia with a platelet count of 125,000/mcL. The diagnosis was further supported by an elevated arterial lactate concentration of 2.4 mmol/L and critically high inflammatory biomarkers, including a procalcitonin concentration of 77.49 ng/mL and a presepsin concentration of 2026 pg/mL. Because the hypotension and the initial lactate level responded promptly to rapid intravascular fluid resuscitation, targeted antimicrobial adjustment, and emergency surgical debridement, the clinical picture was characterized as sepsis with transient hemodynamic instability rather than persistent, refractory septic shock. Microbiological cultures obtained from urinary and wound specimens identified multidrug-resistant Gram-negative organisms, including *Klebsiella pneumoniae*, *Acinetobacter baumannii*, and *Proteus mirabilis*. Blood cultures obtained during the sepsis work-up were positive for *Klebsiella pneumoniae*, confirming bloodstream infection and supporting the diagnosis of severe systemic infection in the context of multiple potential infectious sources. The concordance between bloodstream and local microbiological findings, together with the clinical deterioration and hemodynamic instability, reinforced the interpretation of the episode as mixed-source sepsis involving both urinary and cutaneous reservoirs. Antimicrobial susceptibility testing demonstrated extensive resistance, substantially limiting the available therapeutic options. The *Klebsiella pneumoniae* isolate was resistant to multiple beta-lactams, carbapenems, fluoroquinolones, and trimethoprim/sulfamethoxazole, while susceptibility was retained to amikacin, ceftazidime/avibactam, colistin, gentamicin, and tigecycline. Importantly, the antimicrobial susceptibility profile substantially restricted therapeutic options. Although several agents demonstrated in vitro activity, their clinical applicability was limited by the severity of infection, previous antimicrobial exposure, pharmacological considerations, and the patient’s impaired renal allograft function. The severity of systemic infection, documented *Klebsiella pneumoniae* bacteremia, previous antimicrobial exposure, pharmacological limitations, and the patient’s impaired renal allograft function substantially narrowed the practical therapeutic options. Gentamicin was therefore selected as a salvage therapeutic option because it represented one of the few remaining agents with a realistic probability of achieving effective infection control against the identified MDR pathogen. This decision was not based on a favorable safety profile, but on the urgent need for active antimicrobial therapy in a life-threatening infection. Gentamicin was therefore considered a clinically appropriate option despite its nephrotoxic potential. The *Acinetobacter baumannii* isolate was resistant to ceftazidime, levofloxacin, amikacin, carbapenems, and trimethoprim/sulfamethoxazole, with retained susceptibility to colistin and tigecycline. The detailed antimicrobial susceptibility profiles are presented in Table 2 and Table 3.

The simultaneous presence of symptomatic urinary tract infection and an extensively infected sacral pressure ulcer prevented the unequivocal attribution of bloodstream infection to a single anatomical source. Although blood cultures confirmed *Klebsiella pneumoniae* bacteremia, the exact primary portal of entry could not be definitively established because both the urinary tract and the infected sacral pressure ulcer represented clinically plausible sources of dissemination. The clinical condition was therefore interpreted as mixed-source sepsis of urinary and cutaneous origin, based on the integration of hemodynamic deterioration, clinical findings, sepsis biomarkers, microbiological results, positive blood cultures, and local wound assessment.

The urinary component was considered clinically relevant because systemic manifestations developed during the period in which intermittent self-catheterization had been resumed. Nevertheless, the temporal association did not establish a direct causal relationship, as the patient already had recurrent urinary tract infections and several additional predisposing factors. The infected sacral pressure ulcer simultaneously represented a major potential source of bacterial dissemination and required urgent surgical source control.

Renal assessment was an essential component of the diagnostic and therapeutic decision-making process because the transplanted kidney represented the patient’s sole functioning renal unit. Baseline renal impairment, reflected by a serum creatinine level of 2.28 mg/dL and an estimated glomerular filtration rate of approximately 36.85 mL/min/1.73 m^2^, increased the risk associated with potentially nephrotoxic antimicrobial treatment.

To evaluate for alternative clinical reservoirs of infection, a high-resolution computed tomography (CT) scan of the abdomen and pelvis was performed completely without intravenous contrast exposure to avoid contrast-induced injury to the solitary renal allograft. This was complemented by a renal allograft duplex ultrasound, which demonstrated normal intra-renal resistive indices and excluded mechanical hydronephrosis, perinephric fluid collections, or hidden pelvic abscesses. These findings supported the assessment that the urinary tract and the infected sacral pressure ulcer represented the primary sources of systemic infection.

### 2.4. Therapeutic Intervention

A multidisciplinary team comprising surgeons, infectious disease specialists, nephrologists, and intensive care physicians was assembled. The initial priorities were hemodynamic stabilization, antimicrobial treatment, surgical source control, preservation of renal allograft function, and management of the complex sacral wound. Managing severe sepsis in this patient required an immediate and critical adjustment of his maintenance immunosuppressive regimen to facilitate immune reconstitution while preventing allograft rejection [2,12]. At admission, a renal allograft duplex ultrasound was immediately performed, demonstrating a normal morpho-functional architecture, good global cortical perfusion, and normal intra-renal resistive indices (RI fluctuating between 0.62 and 0.65), while completely ruling out mechanical hydronephrosis, perinephric collections, or major renal vessel thrombosis. Under close transplant nephrology surveillance, the patient’s home immunosuppressive baseline—which originally consisted of Tacrolimus (2 mg bid) with home trough levels targeting 6–8 ng/mL, Mycophenolate Mofetil (MMF, 750 mg bid), and maintenance Prednisone (5 mg/day)—was aggressively modified. Mycophenolate Mofetil was temporarily withheld completely to mitigate severe sepsis-associated bone marrow suppression and facilitate macrophage activation. Chronic maintenance oral Prednisone was switched to stress-dose intravenous Hydrocortisone (100 mg every 8 h) to support vascular tone and hemodynamic stability during the acute septic phase. Concurrently, the maintenance Tacrolimus dose was halved (reduced to 1 mg bid), targeting lower therapeutic trough levels of 4–5 ng/mL (monitored via daily blood FK levels) in order to minimize calcineurin-inhibitor-induced renal afferent arteriolar vasoconstriction in the setting of ongoing acute kidney injury.

The selection of definitive antimicrobial therapy represented the central therapeutic dilemma of this case. Empirical antimicrobial therapy was initially escalated to intravenous meropenem and tigecycline for 48 h. During this empirical window, the patient’s sepsis parameters remained critically severe, characterized by persistent hypotension requiring low-dose vasopressor support and an elevated procalcitonin of 77.49 ng/mL. Following the microbiological confirmation of multidrug-resistant *Klebsiella pneumoniae* bacteremia, the regimen required immediate optimization. Although the isolate showed in vitro susceptibility to tigecycline, tigecycline was deliberately avoided as a primary systemic therapy because it features profound pharmacokinetic limitations in bacteremic sepsis [13,14]. Due to its rapid and extensive tissue distribution, tigecycline exhibits exceptionally low peak serum concentrations (Cmax), carrying an unacceptably high risk of therapeutic failure and increased mortality when utilized as monotherapy for clearing Gram-negative bloodstream infections. Consequently, following multidisciplinary evaluation involving surgical, infectious disease, intensive care, and nephrology teams, intravenous gentamicin was initiated as a culture-directed salvage therapy. The clinical imperative to achieve rapid, concentration-dependent bactericidal clearance of a multidrug-resistant bloodstream infection outweighed the potential risks of aminoglycoside nephrotoxicity [6]. Although the *Klebsiella pneumoniae* isolate demonstrated phenotypic susceptibility to ceftazidime/avibactam, this agent was not immediately available in our hospital pharmacy stock during the acute septic window. Furthermore, gentamicin was preferred over amikacin based on the specific minimum inhibitory concentration profile and the clinical team’s access to a continuously validated, rapid-turnaround infrastructure for automated gentamicin Therapeutic Drug Monitoring (TDM), prioritizing an agent for which precise tracking of pre-dose serum concentrations was available to protect the solitary renal allograft.

**Figure 1:**
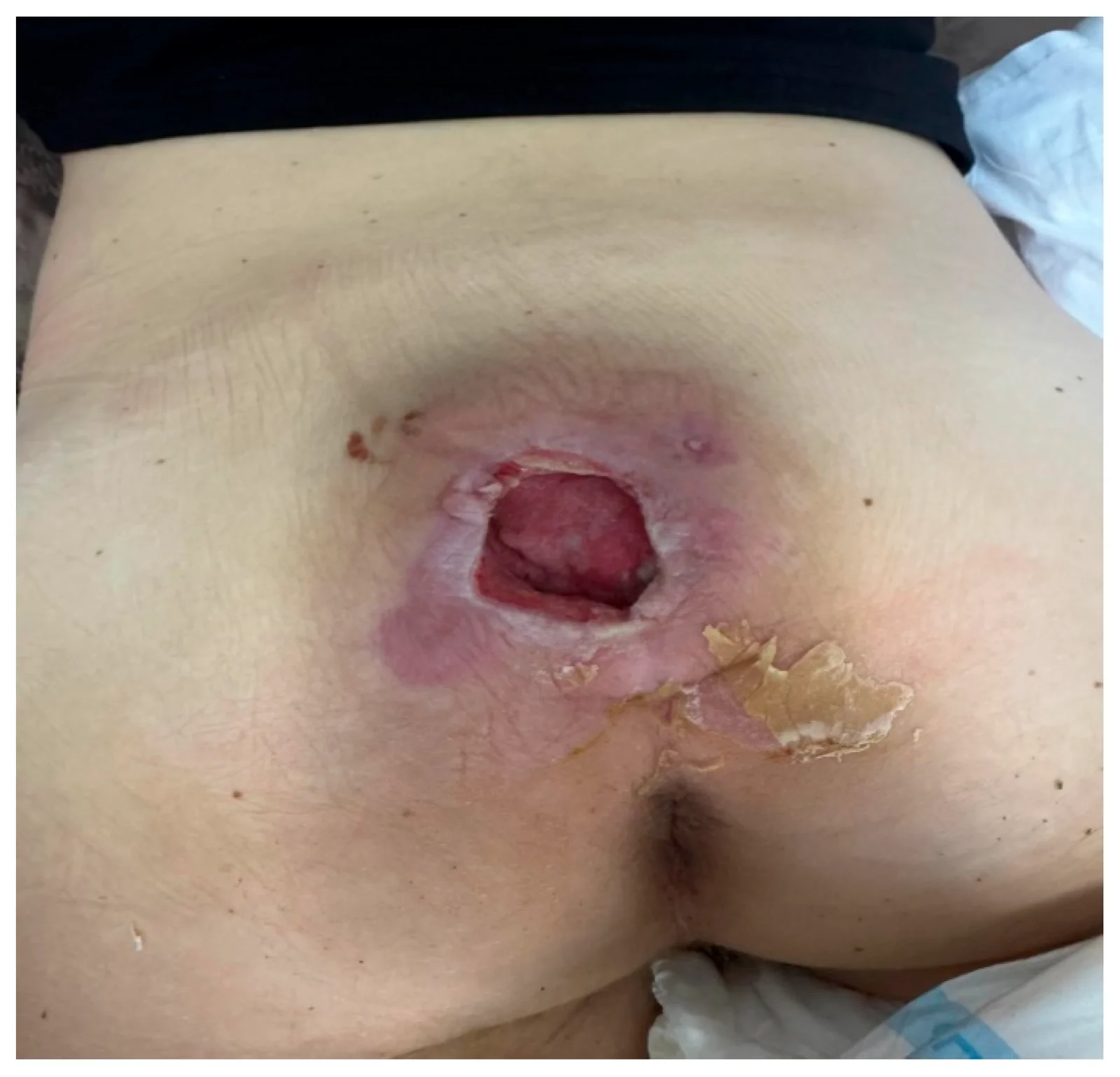
Sacral pressure ulcer prior to initiation of negative pressure wound therapy (NPWT), showing extensive tissue necrosis and local infection.

Emergency surgical intervention was performed, consisting of extensive debridement of the necrotic and infected tissues of the sacral region. The objective was to remove devitalized tissue, reduce the local bacterial burden, facilitate wound drainage, and achieve effective source control (Figure 1).

Following surgical debridement and preparation of the wound bed, negative pressure wound therapy with instillation was initiated using a vacuum-assisted closure system. A sterile polyurethane foam dressing was tailored to the dimensions of the wound and positioned within the defect. The wound was subsequently sealed using an occlusive adhesive drape.

Continuous negative pressure of 100 mmHg was applied through a controlled vacuum system. Dressing changes were performed every four days under sterile conditions. Negative pressure wound therapy was continued until complete control of local infection and development of a healthy granulation tissue bed suitable for definitive surgical reconstruction.

Following microbiological confirmation of multidrug-resistant *Klebsiella pneumoniae* bacteremia, intravenous gentamicin was initiated as a culture-directed salvage therapy in parallel with the local negative pressure wound therapy. Gentamicin was administered at a renal-adjusted dose of 3 mg/kg intravenously every 36 h, which was carefully adapted by the transplant nephrology team to match the patient’s compromised estimated glomerular filtration rate of approximately 36 mL/min/1.73 m^2^. The total duration of aminoglycoside therapy was strictly limited to 7 days.

Therapeutic Drug Monitoring (TDM) was performed rigorously throughout the treatment course; serum gentamicin trough levels were checked exactly 30 min before the administration of every subsequent dose to ensure they remained safely below the nephrotoxicity threshold of 1.0 mcg/mL, successfully preventing proximal tubular drug accumulation. Under strict intravenous hydration protocols to maintain optimal renal cortical perfusion, serial measurements of serum creatinine were monitored daily. The creatinine values fluctuated tightly between 2.1 mg/dL and 2.3 mg/dL during the entire 7 days of gentamicin therapy, showing no precipitous decline, acute tubular deterioration, or any requirement for renal replacement therapy, indicating that allograft preservation was successfully achieved alongside systemic bacterial clearance.

**Figure 2:**
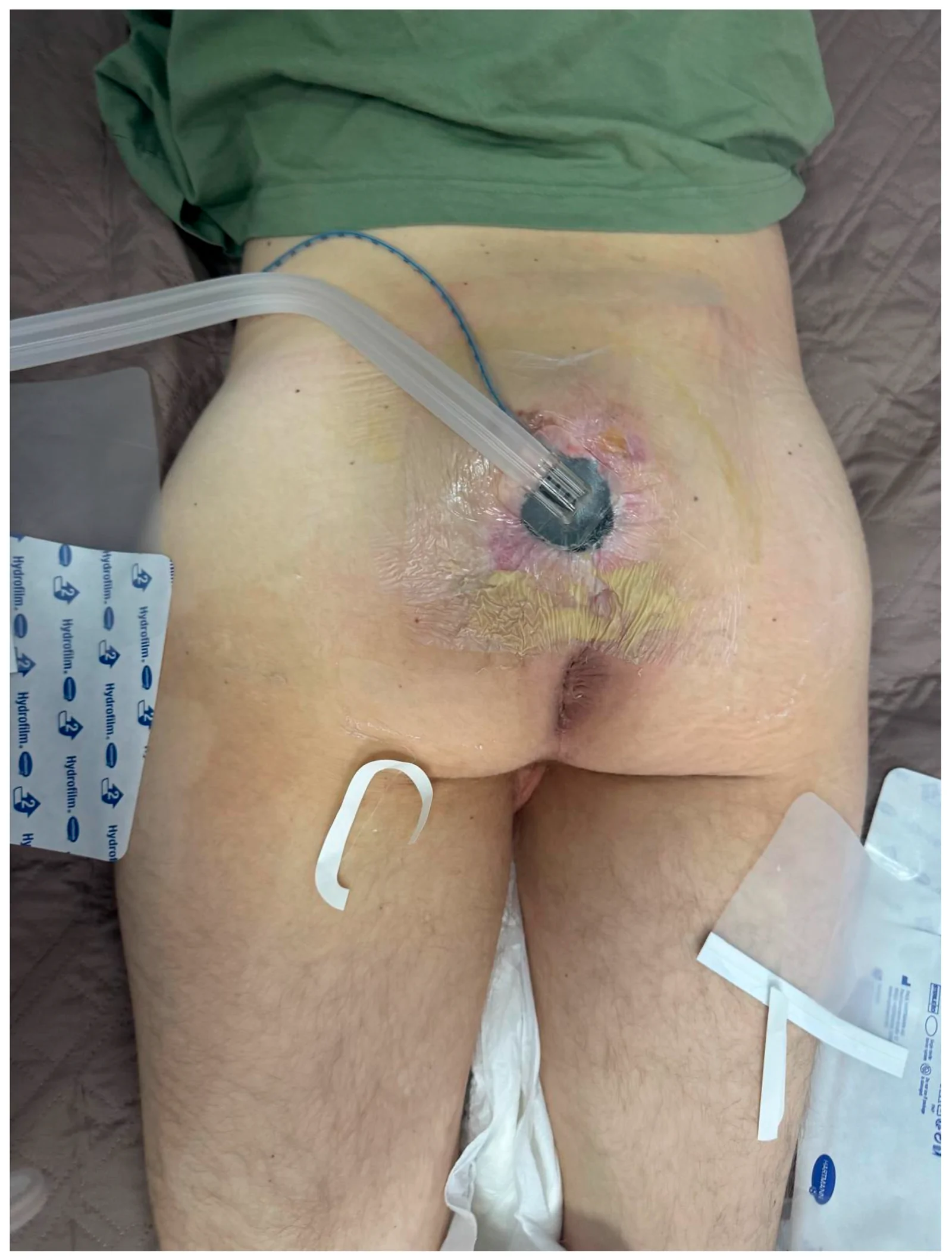
Application of negative pressure wound therapy (NPWT) to the sacral pressure ulcer with instillation.

NPWT was used to facilitate exudate removal, reduce local edema and bacterial burden, and promote the formation of granulation tissue. This approach was consistent with existing recommendations supporting NPWT as an adjunctive treatment for complex and infected wounds, particularly in patients with impaired healing capacity [7,8] (Figure 2).

The selection and deployment of targeted antimicrobial therapy highlighted the central clinical and ethical dilemma of this case, requiring a calculated compromise between immediate, life-saving infection clearance and the long-term preservation of a solitary functioning renal allograft. Given the critical and volatile nature of the multidrug-resistant *Klebsiella pneumoniae* bacteremia, withholding an active bactericidal agent represented an imminent threat to patient survival that far outweighed the secondary, manageable risks of potential aminoglycoside-associated acute kidney injury. In the setting of Gram-negative sepsis, persistent endovascular bacterial seeding triggers severe systemic microvascular endothelial dysfunction, capillary leak, and profound renal hypoperfusion. This cascade can cause irreversible ischemic damage to a solitary transplant unit much faster than a controlled course of target-directed antibiotics.

Consequently, intravenous gentamicin was deployed not as a preferred first-line option, but as a constrained salvage strategy dictated entirely by the exceptionally narrow therapeutic window and the lack of safer alternative agents against carbapenem-resistant Enterobacteriaceae. To successfully navigate this narrow margin of safety and mitigate the potential for acute tubular necrosis, the entire treatment course was executed under a strict, hyper-vigilant nephrological surveillance protocol. This protective framework combined daily serial assessments of serum creatinine, estimated glomerular filtration rate, and hourly urinary output monitoring with continuous therapeutic drug monitoring (TDM) loops, where serum gentamicin trough levels were checked exactly 30 min before the administration of every subsequent 36-h dose to ensure they remained safely below the nephrotoxicity threshold of 1.0 mcg/mL, successfully preventing proximal tubular drug accumulation [6,15]. Furthermore, the clinical team initiated an aggressive, carefully titrated intravenous hydration protocol. This precise fluid strategy was vital to maintain optimal renal cortical blood flow and high distal tubular flow rates, which actively lowered intra-renal aminoglycoside concentrations and successfully shielded the allograft from parenchymal deterioration during systemic bacterial clearance.

### 2.5. Follow-Up and Outcomes

The combined therapeutic strategy, consisting of extensive surgical debridement, negative pressure wound therapy with instillation, culture-directed antimicrobial treatment, and intensive renal monitoring, resulted in progressive clinical improvement and control of the septic episode. Following initiation of targeted antimicrobial therapy, including gentamicin administration for MDR *Klebsiella pneumoniae* bacteremia, the patient demonstrated gradual hemodynamic stabilization, improvement in general condition, and reduction in systemic inflammatory activity.

Serial clinical and laboratory assessments showed resolution of the septic state, with progressive normalization of inflammatory parameters and recovery of clinical stability. Importantly, despite the use of a potentially nephrotoxic antimicrobial agent, no clinically significant deterioration of renal allograft function was observed during treatment. The transplanted kidney remained functional, and kidney replacement therapy was not required.

**Figure 3:**
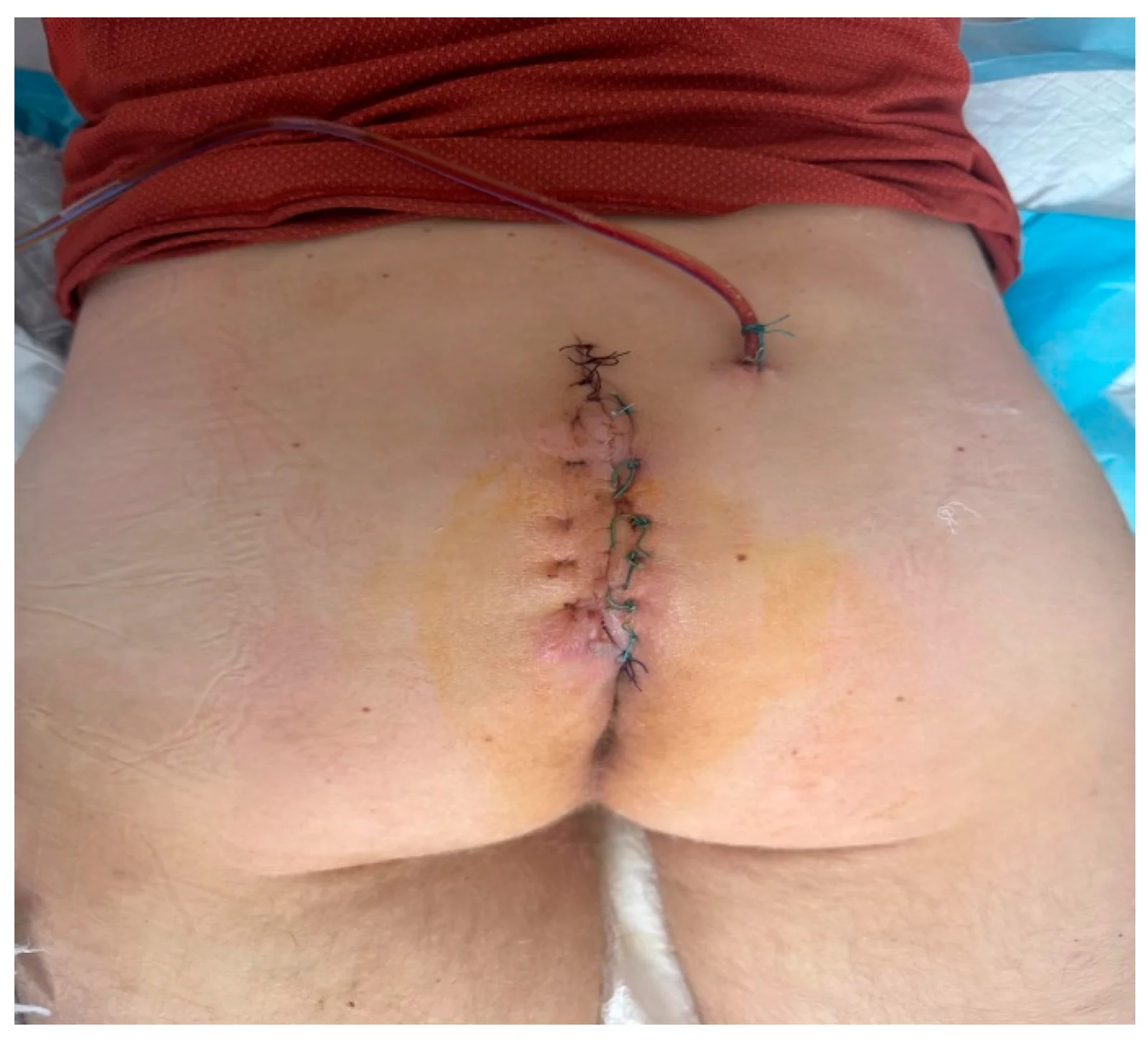
Immediate postoperative appearance following definitive surgical closure of the sacral defect, demonstrating successful reconstruction with satisfactory wound approximation.

Local wound evolution was favorable, with progressive control of infection, reduction in inflammatory changes, and development of a healthy granulation tissue bed suitable for definitive reconstruction. Following adequate local infection control and optimization of the patient’s systemic condition, definitive surgical closure of the sacral defect was performed (Figure 3).

**Figure 4:**
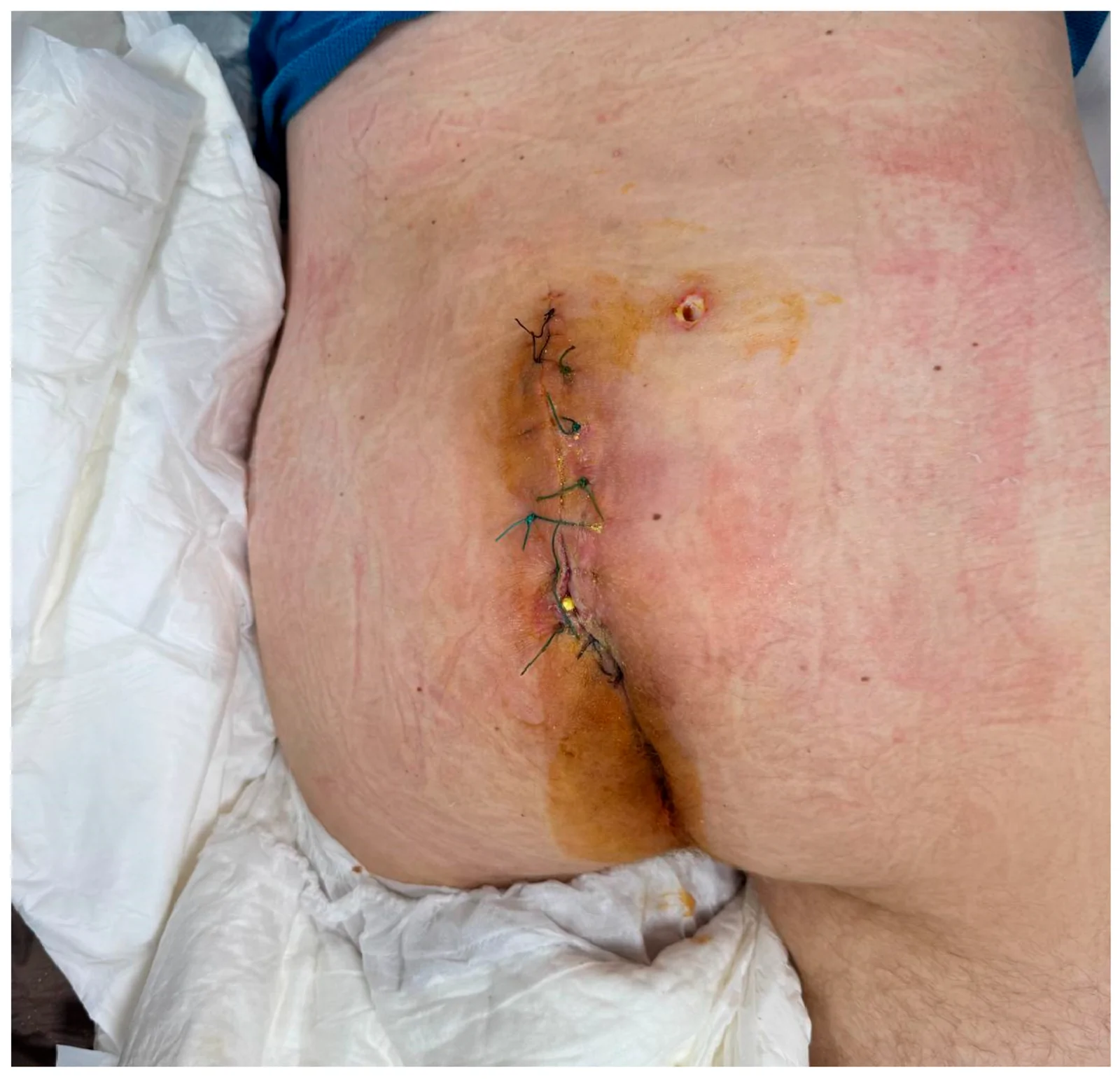
Follow-up postoperative appearance of the sacral region after definitive surgical closure, showing complete wound healing and favorable local evolution.

The postoperative course was favorable, with complete wound healing and no evidence of recurrent local infection. Despite the administration of potentially nephrotoxic antimicrobial therapy, clinically significant deterioration of renal allograft function was not observed during the reported treatment period (Figure 4). 

The patient was ultimately stabilized and transferred for further recovery and rehabilitation. The long-term functional stability of the renal allograft was monitored longitudinally to assess any delayed aminoglycoside-induced proximal tubular toxicity or functional decline [6]. During the 7-day course of intravenous gentamicin salvage therapy, serum creatinine was checked daily, fluctuating tightly between 2.1 mg/dL and 2.3 mg/dL under strict hydration protocols, with no acute uremic spikes. Following successful bacterial clearance and local wound stabilization via negative pressure wound therapy with instillation, the allograft function demonstrated a progressive, favorable trajectory. At the time of hospital discharge on Day 45, the serum creatinine level decreased to 1.45 mg/dL, yielding an eGFR of 54.3 mL/min/1.73 m^2^ via the 2021 CKD-EPI equation. To provide comprehensive longitudinal validation, the patient was closely monitored during scheduled outpatient transplant clinics. At 1 month post-discharge, serum creatinine stabilized at 1.32 mg/dL. Subsequent evaluation at 3 months post-discharge demonstrated a stable renal profile with a creatinine of 1.25 mg/dL. Finally, at the 6-month long-term follow-up appointment, the patient’s serum creatinine returned completely to his pre-septic outpatient baseline of 1.20 mg/dL, with a stable eGFR of 82.3 mL/min/1.73 m^2^, confirming long-term allograft preservation without chronic parenchyma deterioration or late-onset fibrotic decline.

## 3. Discussion

### 3.1. Diagnostic Challenges in an Immunosuppressed Patient with Multiple Potential Infectious Foci

Pressure ulcers remain a major cause of morbidity among patients with spinal cord injury, with reported lifetime prevalence rates exceeding 30% in this population [16]. Their clinical course may be particularly severe in solid-organ transplant recipients, in whom chronic immunosuppression, impaired tissue repair, prolonged hospitalization, and repeated antimicrobial exposure increase the risk of deep soft tissue infection and systemic complications [2,3].

In the present case, several predisposing factors converged, including traumatic paraplegia, prolonged immobilization, chronic immunosuppressive therapy, neurogenic lower urinary tract dysfunction, recurrent urinary tract infections, and progressive infection of a sacral pressure ulcer. Immunosuppressive therapy may impair both innate and adaptive immune responses, reducing the host’s capacity to contain bacterial proliferation and potentially attenuating or modifying the clinical manifestations of infection [2,12]. Moreover, impaired inflammatory signaling, angiogenesis, fibroblast activity, and collagen deposition may delay wound healing and facilitate the progression of pressure injuries toward extensive soft tissue destruction [17].

#### 3.1.1. The Challenge of Identifying the Primary Source of Infection

The principal diagnostic difficulty was the identification and characterization of systemic infection in the presence of two concomitant potential infectious sources. Recurrent urinary tract infections associated with neurogenic bladder dysfunction and intermittent self-catheterization coexisted with an extensively infected sacral pressure ulcer containing necrotic tissue. Under these circumstances, neither the urinary tract nor the wound could be unequivocally established as the sole source of sepsis.

This case combined several established risk factors for severe infection: chronic immunosuppression, traumatic paraplegia with prolonged immobilization, neurogenic bladder requiring urinary instrumentation, recurrent urinary tract infection, and an infected sacral pressure ulcer. Their coexistence complicated both source attribution and antimicrobial decision-making.

The temporal relationship between the resumption of intermittent self-catheterization and the development of systemic urinary manifestations suggested that repeated urinary tract instrumentation may have contributed to the infectious episode. However, a direct causal relationship cannot be established, because the patient had already experienced recurrent urinary tract infections and presented several additional predisposing factors, including chronic immunosuppression, incomplete bladder emptying, previous antimicrobial exposure, and prolonged hospitalization.

Clean intermittent catheterization is generally preferred to prolonged indwelling catheterization for long-term bladder emptying in patients with neurogenic lower urinary tract dysfunction. Nevertheless, repeated instrumentation, urinary stasis, previous infections, immunosuppression, and colonization with resistant organisms may facilitate progression from asymptomatic bacteriuria to symptomatic or systemic infection [18,19]. In kidney transplant recipients, MDR Gram-negative urinary infections complicated by bacteremia represent a significant therapeutic challenge because of their association with prior antimicrobial exposure, nosocomial acquisition, and limited antimicrobial options. In such patients, microbiological findings must be correlated with the clinical context and with potentially correctable abnormalities, including urinary retention, obstruction, inadequate drainage, infected urinary devices, hydronephrosis, or renal and perirenal collections [18,20].

The sacral pressure ulcer simultaneously represented a major potential source of bacterial dissemination. Extensive necrosis, progressive local inflammation, increasing wound dimensions, and deep soft tissue involvement supported the diagnosis of clinically significant wound infection rather than superficial colonization. The coexistence of several infectious foci is clinically relevant, because polymicrobial infection and multiple simultaneous sources have been associated with an increased risk of septic progression and adverse outcomes [21].

#### 3.1.2. The Role of Renal Function Assessment

The diagnostic process was further complicated by pre-existing renal allograft dysfunction. Renal impairment during severe infection may result from several interacting mechanisms, including sepsis-related hypoperfusion, altered intravascular volume, urinary tract dysfunction, previous nephrotoxic exposure, and antimicrobial-associated renal injury. Renal allograft assessment therefore constituted an essential component of both the diagnostic evaluation and therapeutic decision-making.

Overall, this case illustrates that the diagnosis of sepsis in an immunosuppressed kidney transplant recipient cannot rely on a single clinical, biochemical, or microbiological parameter. This diagnostic complexity is particularly relevant in solid-organ transplant recipients, in whom bloodstream infections may present with atypical manifestations and require integration of microbiological data, clinical evolution, and identification of potential infectious reservoirs [22]. It requires integrated assessment of the clinical trajectory, hemodynamic status, inflammatory biomarkers, culture results, antimicrobial susceptibility profiles, wound characteristics, urinary tract function, and renal allograft status.

### 3.2. Multidrug-Resistant Infection and Antimicrobial Decision-Making

A defining feature of this case was the identification of multidrug-resistant Gram-negative organisms, particularly *Klebsiella pneumoniae* and *Acinetobacter baumannii*. The global dissemination of carbapenemase-producing *Klebsiella pneumoniae* has significantly reduced therapeutic options and represents a major challenge in healthcare-associated infections [23]. These pathogens are major causes of healthcare-associated infection and are recognized as critical-priority organisms because of their extensive resistance profiles and the limited availability of consistently effective antimicrobial agents [5]. Carbapenem-resistant *Klebsiella pneumoniae* and *Acinetobacter baumannii* infections are associated with substantial morbidity and mortality, particularly among critically ill and immunocompromised patients [9,24]. Kidney transplant recipients are particularly vulnerable to MDR bacterial infections because of chronic immunosuppression, repeated healthcare exposure, previous antimicrobial therapy, and frequent invasive procedures, factors that may significantly influence both microbiological profiles and therapeutic outcomes [25].

The antimicrobial susceptibility profiles demonstrated resistance to multiple beta-lactams, carbapenems, fluoroquinolones, and other commonly used agents. In particular, the *Klebsiella pneumoniae* isolate showed resistance to the majority of commonly used antimicrobial classes, leaving only limited active therapeutic alternatives. This markedly narrowed the therapeutic window and necessitated consideration of gentamicin despite the increased risk of nephrotoxicity. This pattern considerably restricted therapeutic options and required individualized interpretation of in vitro susceptibility in relation to the presumed infection sites, previous antimicrobial exposure, expected drug distribution, toxicity profile, and baseline renal function.

Current evidence regarding carbapenem-resistant Enterobacterales emphasizes that therapeutic decisions must carefully weigh the severity of infection, antimicrobial resistance patterns, and patient-specific factors, as in vitro susceptibility alone may not fully predict clinical success [14]. In a critically ill kidney transplant recipient with an active bloodstream infection, patient-specific factors dictate a strict clinical hierarchy. The immediate imperative to achieve rapid and reliable bactericidal clearance of a life-threatening sepsis must take precedence over the secondary risk of potential drug-induced nephrotoxicity, given that an uncontrolled endovascular infection carries an imminent risk of mortality before long-term graft preservation can even be considered.

Our therapeutic approach closely aligns with the management algorithms outlined in the recent Infectious Diseases Society of America (IDSA) and European Society of Clinical Microbiology and Infectious Diseases (ESCMID) guidelines for resistant Gram-negative infections [13,14]. Current international consensus emphasizes that for severe bacteremia or sepsis caused by carbapenem-resistant Enterobacteriaceae (CRE), treatment selection must be strictly directed by clinical-pharmacokinetic viability and the localized anatomical site involved. In scenarios where novel beta-lactam/beta-lactamase inhibitor combinations are constrained by immediate institutional availability, guidelines accept the targeted deployment of concentration-dependent bactericidal agents like aminoglycosides as vital definitive or salvage regimens, provided that extended-interval dosing and rigorous monitoring are actively maintained.

In this patient, the therapeutic selection required a calculated compromise between immediate infection control and allograft preservation. While colistin was associated with a clinically prohibitive risk of severe, irreversible nephrotoxicity in a solitary renal allograft [26], tigecycline presented profound pharmacokinetic limitations in severe systemic bloodstream infections [13,14] due to its rapid tissue penetration and low peak serum concentrations (Cmax), which correlates with treatment failure when utilized as monotherapy for clearing Gram-negative bloodstream infections [15]. Consequently, gentamicin was retained as the most viable salvage strategy. Rather than a preferred first-line strategy, this aminoglycoside course represented a constrained option dictated by the narrow therapeutic window, executed under strict nephrological surveillance and pre-dose therapeutic drug monitoring loops to prevent proximal tubular drug accumulation. Consequently, each potentially active agent had to be evaluated in relation to both its anticipated clinical efficacy and its toxicity.

Aminoglycosides provide rapid, concentration-dependent bactericidal activity against susceptible Gram-negative organisms and may remain clinically valuable when antimicrobial resistance limits the use of less toxic alternatives [27]. Their administration in kidney transplant recipients is nevertheless particularly challenging because aminoglycoside-associated proximal tubular injury may lead to acute kidney injury and compromise graft function [6,28]. The risk of toxicity is influenced by cumulative exposure, duration of treatment, and patient-specific factors, emphasizing the importance of therapeutic monitoring [15].

In the present case, gentamicin was selected following multidisciplinary evaluation because it represented one of the few clinically viable active options against the MDR *Klebsiella pneumoniae* isolate. This decision represented the central therapeutic dilemma of the case, as gentamicin was not selected as a preferred antimicrobial agent but rather as a constrained salvage option imposed by the MDR resistance profile and the absence of equally effective, less nephrotoxic alternatives. Importantly, gentamicin was not selected as a preferred first-line strategy, but rather as a salvage option dictated by the limited number of active agents available against the MDR *Klebsiella pneumoniae* isolate and the severity of the ongoing systemic infection. Withholding an active antimicrobial agent carried an immediate risk of persistent infection, uncontrolled sepsis, multiorgan dysfunction, and death, whereas gentamicin administration exposed the patient’s sole functioning renal allograft to the possibility of acute and potentially irreversible nephrotoxicity.

In life-threatening infection, aminoglycoside therapy is not absolutely contraindicated when it represents a clinically appropriate active option. Its use should nevertheless be individualized, adjusted to renal function, limited to the shortest effective duration, and accompanied by close monitoring of renal function and urinary output [6,20]. Whenever possible, concomitant nephrotoxic agents should be avoided, intravascular volume and renal perfusion should be optimized, and the immunosuppressive regimen should be reassessed jointly by infectious disease and transplant nephrology specialists.

The favorable clinical evolution observed in this patient supports the principle that antimicrobial nephrotoxicity must be evaluated in the context of the immediate risk posed by uncontrolled sepsis. Preservation of renal allograft function remained an important therapeutic objective; however, rapid infection control and patient survival necessarily represented the initial priorities.

### 3.3. Renal Allograft Preservation and the Role of Transplant Nephrectomy

The renal allograft represented the patient’s only functioning renal unit, making preservation of graft function a major therapeutic objective. Nevertheless, severe infection and the need for potentially nephrotoxic antimicrobial treatment raised concern regarding irreversible graft dysfunction and the possible requirement for kidney replacement therapy [29].

The absence of clinically significant deterioration in graft function during the reported treatment period supports the value of individualized risk–benefit assessment, adequate urinary drainage, careful antimicrobial selection, and close nephrological surveillance. This favorable outcome should not be interpreted as evidence that aminoglycosides are devoid of risk in kidney transplant recipients. Rather, it illustrates that potentially nephrotoxic therapy may be justified in selected life-threatening circumstances when treatment is carefully monitored and safer clinically effective alternatives are limited.

### 3.4. Surgical Source Control and Negative Pressure Wound Therapy

Systemic antimicrobial therapy alone is often insufficient when extensive necrotic tissue and a persistent infected wound remain present. Early and adequate surgical source control is therefore a fundamental component of sepsis management. In infected pressure ulcers, debridement removes devitalized tissue, decreases the local bacterial burden, facilitates drainage, improves assessment of wound depth and extent, and creates conditions favorable for subsequent healing [30,31].

In the present case, extensive surgical debridement was required because of progressive sacral wound infection, tissue necrosis, and deep soft tissue involvement. Debridement was followed by negative pressure wound therapy with instillation, which was used as an adjunct to systemic antimicrobial therapy rather than as a substitute for adequate source control.

NPWT contributes to wound management by promoting removal of exudate, reducing local edema, stabilizing the wound environment, and stimulating granulation tissue formation. Recent evidence supports its application in complex wounds by improving local wound conditions, facilitating granulation tissue development, and serving as an adjunctive strategy when conventional wound management is insufficient [7,8,32]. Its use has been described in complex and infected wounds, including wounds healing by secondary intention and wounds occurring in patients with impaired healing capacity [7,8].

The favorable local evolution, characterized by progressive infection control and the formation of healthy granulation tissue, permitted subsequent definitive surgical closure. This staged strategy is particularly relevant in clinically unstable and immunosuppressed patients. Immediate definitive closure in the presence of uncontrolled infection, persistent necrosis, or inadequate wound-bed preparation could increase the risk of dehiscence, persistent infection, or recurrent tissue breakdown.

The outcome observed in this patient cannot be attributed to NPWT alone. It resulted from the combined effects of extensive debridement, systemic culture-directed antimicrobial therapy, urinary management, hemodynamic stabilization, renal monitoring, repeated wound assessment, and subsequent definitive closure. Similar multimodal approaches integrating vacuum-assisted techniques with aggressive source control have been described in severe septic conditions, emphasizing the role of negative pressure therapies as adjunctive strategies rather than substitutes for definitive infection control [33]. NPWT should therefore be interpreted as one component of a broader multidisciplinary treatment strategy.

### 3.5. Clinical Relevance and Contribution to the Literature

The clinical uniqueness of this case lies in the complex convergence of severe infectious risks: a kidney transplant recipient with a solitary functional allograft, complete traumatic paraplegia with neurogenic bladder dysfunction, and an infected stage IV sacral ulcer, culminating in critical multidrug-resistant (MDR) *Klebsiella pneumoniae* bacteremia. While separate studies document MDR urinary tract infections or negative pressure wound therapy (NPWT) execution in spinal cord injury patients, evidence addressing their simultaneous management in an active solid-organ transplant setting remains exceptionally scarce.

A primary learning point from this case is the validation of aggressive, staged multidisciplinary source control over conservative antibiotic escalation alone. The chronological transition of an unstable, infected wound bed into a clean, reconstructible area via customized NPWT with instillation highlights the necessity of absolute local source control before definitive flap reconstruction can succeed. Furthermore, the case underscores a critical clinical decision-making paradigm: in life-threatening sepsis, the immediate requirement for high-efficacy bactericidal therapy against a documented bloodstream infection must take precedence over the secondary risk of antimicrobial-induced organ toxicity. When managed through tight multidisciplinary surveillance and therapeutic drug monitoring (TDM), safe allograft preservation is achievable even during a high-risk nephrotoxic salvage course.

Rather than definitively attributing the bacteremia to a single anatomical source—a task limited by the coexistence of two highly plausible reservoirs (the urinary tract and the sacral wound)—this case demonstrates how clinicians must integrate overlapping regional symptom peaks and phenotypic antibiograms to drive survival. This clinical timeline provides a practical reference framework for managing extreme multi-source infections in vulnerable, immunosuppressed patient cohorts, emphasizing that successful outcomes rely on the coordinated implementation of cross-disciplinary interventions rather than a single therapeutic agent.

### 3.6. Limitations

This report has several limitations. As a single-patient observation, it cannot establish causal relationships or support generalized therapeutic recommendations. A primary limitation is the absence of molecular and genotypic characterization of the isolated multidrug-resistant (MDR) pathogens. Because advanced molecular assays (such as PCR for specific carbapenemase genes, pulsed-field gel electrophoresis, or whole-genome sequencing) were not available, we could not definitively identify the specific beta-lactamase or carbapenemase families driving the resistance profiles. Furthermore, the lack of genomic typing prevents us from establishing absolute clonal identicalness between the K. pneumoniae strain isolated in the blood and those recovered from the urine and wound beds. Consequently, the dual-source origin of the bacteremia remains an inferred clinical diagnosis based on concordant phenotypic antibiograms and overlapping regional symptom peaks, rather than a molecularly proven epidemiological link. This limits our comprehensive understanding of the precise resistance mechanisms involved and masks their potential implications for long-term epidemiological tracking and future targeted treatment strategies, highlighting the ongoing necessity for expanded access to molecular microbiological testing in complex transplant care.

## 4. Conclusions

This case illustrates the complexity of managing severe multidrug-resistant infection in a kidney transplant recipient with traumatic paraplegia, neurogenic bladder dysfunction, and an infected sacral pressure ulcer. The coexistence of urinary and cutaneous infectious sources, together with the possibility of colonization by resistant organisms, required integration of clinical evolution, hemodynamic status, inflammatory biomarkers, microbiological findings, antimicrobial susceptibility testing, and direct wound assessment.

Successful management required a staged and multidisciplinary strategy combining surgical source control, negative pressure wound therapy with instillation, definitive wound closure, individualized antimicrobial therapy, and continuous renal allograft monitoring.

The use of gentamicin represented a critical therapeutic dilemma because of the risk of nephrotoxicity in a patient with a solitary functioning renal allograft. However, in the context of life-threatening MDR sepsis and limited effective alternatives, the potential benefit of achieving infection control outweighed the anticipated risk when therapy was administered under strict nephrological surveillance.

This case highlights that in complex transplant-associated infections, treatment decisions must be individualized according to the balance between immediate mortality risk from uncontrolled infection and potential treatment-related organ toxicity. Preservation of renal graft function and effective infection control should be considered complementary objectives achievable through coordinated multidisciplinary care.

## Figures and Tables

**Table 1:** Trends in hematological, renal, and inflammatory laboratory parameters during hospitalization. Abbreviations: eGFR, estimated glomerular filtration rate; CRP, C-reactive protein.

Clinical Phase & Timeline	Key Parameters & Diagnostic Findings	Therapeutic Adjustments & Interventions
Phase 1: Admission (Hospital Day 1)	• Hemoglobin: 8.3 g/dL • Hematocrit: 25.7% • Serum Creatinine: 2.28 mg/dL (AKI Stage 2) • eGFR: 36.85 mL/min/1.73 m^2^ (via 2021 CKD-EPI) • CRP: 5.2 mg/L|Procalcitonin: <0.05 ng/mL • Baseline stable creatinine verified at 1.20 mg/dL over past 6 months.	• Fluid resuscitation for post-traumatic hypovolemia. • Temporary indwelling Foley catheter placed strictly for 72 h perioperatively, then shifted back to maintenance clean intermittent self-catheterization. • Routine pressure injury prevention protocol initiated.
Phase 2: Chronic Evolution (Hospital Days 2–60)	• Progressive development of infected stage IV sacral pressure injury with extensive tissue necrosis. • Recurrent urinary tract infections (UTIs) secondary to neurogenic bladder dysfunction. • Secondary Clostridioides difficile infection (CDI) confirmed via positive fecal stool PCR assay for toxins A and B.	• Multiple empirical courses of Ceftriaxone and Ciprofloxacin for recurrent UTIs. • CDI successfully eradicated with a 10-day course of oral Vancomycin (125 mg every 6 h). • Optimization with customized hyperproteic nutrition support (zinc, arginine, antioxidants).
Phase 3: Acute Sepsis (Hospital Day 61)	• True Bacterial Sepsis documented via Sepsis-3 criteria (Acute SOFA = 5 points): Cardiovascular (2), Renal (2), Metabolic/Hematological (1). • Hemoglobin: 5.4 g/dL|Hematocrit: 15.9% • Serum Creatinine: 2.28 mg/dL • CRP: 31.2 mg/L|Procalcitonin: 77.49 ng/mL • Presepsin: 2026 pg/mL|Arterial Lactate: 2.4 mmol/L • High-resolution abdominal/pelvic CT scan performed completely without intravenous contrast + renal graft duplex ultrasound (confirmed normal resistive indices, RI 0.62–0.65). • Blood cultures return positive for carbapenem-resistant *Klebsiella pneumoniae*.	• Maintenance transplant immunosuppression optimized: MMF temporarily withheld to reduce overall immunosuppressive burden, oral Prednisone switched to IV stress-dose Hydrocortisone (100 mg every 8 h), and Tacrolimus dose halved to target a lower trough of 4–5 ng/mL. • Empiric therapy shifted to Meropenem + Tigecycline for 48 h, then optimized to targeted intravenous Gentamicin (3 mg/kg every 36 h for a strict duration of 7 days) due to institutional availability and superior TDM infrastructure. • Emergency surgical wound debridement followed immediately by continuous Negative Pressure Wound Therapy (NPWT) with instillation at 100 mmHg.
Phase 4: Discharge & Follow-Up (Hospital Day 45 & 6 Months Outpatient)	Day 45 (Discharge): • Hemoglobin: 11.2 g/dL|Hematocrit: 34.1% • Serum Creatinine: 1.45 mg/dL|eGFR: 54.3 mL/min/1.73 m^2^ • CRP: <5.0 mg/L|Procalcitonin: 0.12 ng/mL 6 Months Post-Discharge (Outpatient Clinic): • Serum Creatinine: 1.20 mg/dL|eGFR: 82.3 mL/min/1.73 m^2^	• Diligent Therapeutic Drug Monitoring (TDM) checked exactly 30 min before each Gentamicin dose, maintaining trough levels safely below 1.0 mcg/mL. • Successful delayed definitive surgical closure of the sacral defect following local infection clearance. • Long-term longitudinal validation confirms complete preservation of the solitary renal allograft unit returning entirely to its pre-injury outpatient baseline.

**Table 2:** Antimicrobial susceptibility profile of *Klebsiella pneumoniae*.

Antibiotic	MIC	Interpretation
Amoxicillin/Clavulanic Acid	>32/2	Resistant
Amikacin	≤2	**Sensitive**
Ampicillin/Sulbactam	>32	Resistant
Ceftazidime	>16	Resistant
Ceftazidime/Avibactam	≤8	**Sensitive**
Cefepime	>32	Resistant
Ceftriaxone	>8	Resistant
Ciprofloxacin	<0.5	Resistant
Colistin	≤0.5	**Sensitive**
Cefotaxime	>4	Resistant
Cefuroxime	>4	Resistant
Ertapenem	>8	Resistant
Gentamicin	≤0.5	**Sensitive**
Imipenem	>4	Resistant
Imipenem/Relebactam	>2/4	Resistant
Levofloxacin	>4	Resistant
Meropenem	>32	Resistant
Piperacillin/Tazobactam	>16/4	Resistant
Trimethoprim/Sulfamethoxazole	>4/76	Resistant
Tobramycin	>4	Resistant
Tigecycline	≤0.5	**Sensitive**

**Table 3:** Antimicrobial susceptibility profile of *Acinetobacter baumannii*.

Antibiotic	MIC	Interpretation
Ceftazidime	>2	Resistant
Levofloxacin	>2	Resistant
Amikacin	>32	Resistant
Colistin	≤1	**Sensitive**
Trimethoprim/Sulfamethoxazole	>4/76	Resistant
Imipenem	>8	Resistant
Meropenem	>8	Resistant
Tigecycline	≤0.25	**Sensitive**

## Data Availability

The original contributions presented in this study are included in the article. Further inquiries can be directed to the corresponding author.
